# Successful Case of Laparotomy

**Published:** 1890-04

**Authors:** R. E. Moss

**Affiliations:** Mexia


For Daniel's Texas Medical Journal:

### SUCCESSFUL CASE OF LAPAROTOMY.

R. E. MOSS, M. D., MEXIA.

PATIENT Mrs. M, æt 58, mother of five children, always healthy and never noticed any abdominal enlargement until about two years ago, she noticed a small hard mass in the median line just above the pubic bone.

In never gave any pain, but was rapidly increasing in size, which caused her to consult me on February 3, 1890.

After being told that an operation was the only plan of relief; she was eager to have it done next day. I postponed it until the 6th, in order to prepare the patient, and also the room.

After making an incision through the median line and exposing the cyst wall, we tapped it, drawing off a gallon and a half of dark thick grumous fluid, but found we had a multilocular cyst to deal with. The other four compartments contained a straw colored fluid.

The left broad ligament, tube and ovary were ligated and removed; being so intimately connected with the tumor that separation would have been impossible, and the ovary proved to be cystic.

The tumor seemed to grow out of the posterior wall of the uterus, and had no real pedicle, which necessitated ligating it in sections.

After the mass was removed the abdominal cavity was freely flushed with hot bi-chloride water and mopped dry with clean carbolized sponges.

The opening was closed with silver wire, and dressed with dry iodoform wool cotton, kept in place with a flannel roller. She was put to bed in good condition and made a nice recovery without a single unfavorable sympton.

I report this just to add another successful case to the list of Texas operations, and as an advocate of completely closing the abdominal incision to keep out the "little pestiferous bugs."

If drainage becomes necessary, secure it through an opening into Douglas's pouch.

